# Cerebral Vasospasm in Traumatic Brain Injury

**DOI:** 10.1155/2013/415813

**Published:** 2013-06-19

**Authors:** Daniel R. Kramer, Jesse L. Winer, B. A. Matthew Pease, Arun P. Amar, William J. Mack

**Affiliations:** Department of Neurosurgery, University of Southern California, 1200 North State Street, Suite 3300, Los Angeles, CA 90033, USA

## Abstract

Vasospasm following traumatic brain injury (TBI) may dramatically affect the neurological and functional recovery of a vulnerable patient population. While the reported incidence of traumatic vasospasm ranges from 19%–68%, the true incidence remains unknown due to variability in protocols for its detection. Only 3.9%–16.6% of patients exhibit clinical deficits. Compared to vasospasm resulting from aneurysmal SAH (aSAH), the onset occurs earlier and the duration is shorter. Overall, the clinical course tends to be milder, although extreme cases may occur. Traumatic vasospasm can occur in the absence of subarachnoid hemorrhage. Surveillance transcranial Doppler ultrasonography (TCD) has been utilized to monitor for radiographic vasospasm following TBI. However, effective treatment modalities remain limited. Hypertension and hypervolemia, the mainstays of treatment of vasospasm associated with aSAH, must be used judiciously in TBI patients, and calcium-channel blockers have offered mixed clinical results. Currently, the paucity of large prospective cohort studies and level-one data limits the ability to form evidence-based recommendations regarding the diagnosis and management of vasospasm associated with TBI.

## 1. Introduction

Traumatic brain injury (TBI) bears a heavy societal burden [1]. With an incidence rate of approximately 1.5 million new cases per year, TBI is the leading cause of death in the USA between the ages of one and forty-five [1]. Primary TBI management focuses on patient stabilization and treatment of elevated intracranial pressure (ICP). Surgical decompression and/or clot evacuation can decrease mass effect and cerebral edema, thus mitigating progressive neurological decline [2, 3]. The frequency of SAH following head trauma is estimated at 39%–65% [4–9], and its presence is an independent predictor of poor functional outcome [4, 7, 10, 11]. Severity of hemorrhage on CT scan has been shown to correlate with clinical status [12]. Although significant morbidity and mortality are attributable to the inciting trauma, deleterious sequelae of secondary injury are considerable and remain a critical focus of medical therapies. 

Vasospasm is a delayed, secondary consequence that can profoundly impact neurological recovery and functional outcome after TBI. Although vasospasm may result from traumatic subarachnoid hemorrhage (tSAH), other mechanisms such as blast-induced neurotrauma are increasingly recognized as causative factors [13–16]. This paper reviews the epidemiology, diagnosis, pathophysiology, prevention, and treatment of vasospasm secondary to traumatic brain injury.

## 2. Vasospasm Background

The vast preponderance of current knowledge regarding cerebral vasospasm derives from the study of aneurysmal subarachnoid hemorrhage (aSAH). Symptomatic (clinical) vasospasm occurs in 20%–30% of aSAH patients and accounts for 50% of the posthemorrhage mortality in those who survive to treatment [17–20]. Pathophysiology is poorly understood, and management strategies are only moderately effective. Approximately half of all aSAH patients with vasospasm develop delayed cerebral ischemia, which is associated with a 20% incidence of significant morbidity and/or mortality [19, 21, 22]. Although, recently, advances in medical management have decreased the morbidity and mortality from vasospasm, it remains the most hazardous complication of aSAH [23, 24]. Continued research efforts focus on novel diagnostic markers and putative therapeutic agents. 

While head trauma is the most common cause of bleeding in the subarachnoid space [25], the literature is sparse and the data is minimal regarding vasospasm in this setting [4]. Existing knowledge is derived, primarily, from small case series and subsets of patient cohorts within the SAH literature.

## 3. Epidemiology of Vasospasm in Traumatic Subarachnoid Hemorrhage

Cerebral vasospasm following head trauma is characterized by an earlier onset than that of aneurysmal subarachnoid hemorrhage (see Table 1). Vasospasm tends to occur between days four and fourteen following aSAH [19], and it rarely appears prior to day three [8, 26]. By contrast, studies have suggested that 25% of vasospasm secondary to tSAH occurs by day three [8], with the onset regularly noted in the first two days after injury [9, 10, 27–30]. Temporal latency of greatest vessel narrowing is also more rapid in onset. Maximal TCD velocities in tSAH are reported on postinjury days 5–7, whereas peak values are documented on days 5–14 after aSAH [19, 27]. The duration of vasospasm in tSAH is often shorter than that of aSAH. Vasospasm tends to resolve completely within fourteen days of traumatic brain injury, although cases lasting up to 30 days have been reported [8, 13, 26, 27]. Post-traumatic vasospasm in the absence of tSAH, generally, has an even briefer duration. Martin et al. documented an average time course of 1.25 days, implicating an alternative pathophysiology [31].

The initial hemorrhagic insult and resultant blood pattern likely account for the unique time course and trajectory of vasospasm following tSAH. For instance, tSAH is often distributed over the cerebral convexity, while aSAH is typically confined to the basal cisterns. Regional clearance of cerebrospinal fluid might differ between these two surfaces of the brain. Furthermore, the arteries that course within the subarachnoid space of these two regions differ in caliber, flow, and collateral circulation. These and other variances might explain the differential incidence and clinical course of vasospasm in aSAH versus tSAH. A direct comparison between tSAH and aSAH patients conducted by Fukuda et al. revealed that trauma was associated with more diffuse subarachnoid and intracranial blood which, in turn, resolved more rapidly. Aneurysmal SAH, on the other hand, resulted in increased regions of hypodensity related to discrete vascular territories on CT scan [12]. Taken together, these time course and radiographic findings suggest a more benign course for vasospasm secondary to tSAH.

There is a direct correlation between quantity of blood in the subarachnoid cisterns and risk of vasospasm following aSAH [26]. Although a similar association has been reported in tSAH [8, 40], the relationship is less concrete. Gaetani et al. noted that, while the Fisher grade was not correlated with clinical presentation following traumatic head injury, it was predictive of Glasgow outcome score at 6 months [41]. However, causal inference is problematic due to the complex, multifactorial sequelae of the injuries associated with TBI. Zubkov et al. reported an increased incidence of vasospasm (measured by TCD) in TBI patients with traumatic epidural and subdural hematomas, but not with intracerebral or intraventricular hemorrhage. However, the sample size in the study was relatively small [8].

The pathophysiology of vasospasm following TBI does not depend entirely upon the presence of blood in the subarachnoid space. This indistinct relationship is underscored by reports of radiographic vasospasm in 10%–30% of TBI patients lacking evidence of subarachnoid hemorrhage [8, 11, 28, 42]. Oertel et al. propose that low GCS could be a greater relative risk factor for vasospasm than cisternal blood [28]. Although radiographic vasospasm following TBI appears to be common, associated delayed neurological ischemic deficits are even less predictable than those observed in aSAH. Only 3.9%–16.6% of tSAH patients with delayed radiographic or TCD-diagnosed cerebral vasospasm develop referable neurological deficits [8, 11, 30, 39], compared to 17%–40% of patients with vasospasm secondary to aSAH [26] (see Table 1). It must be noted, however, that the ability to detect clinically significant deficits in TBI patients is often a challenge due to the decreased level of consciousness secondary to the confounding factors and alternate mechanisms of injury [28]. Development of cerebral ischemia can serve as a surrogate marker of pathology. A study by Chan et al. demonstrated a 17% rate of cerebral infarction on CT scans among TBI patients with associated vasospasm. Head trauma patients without radiographic vasospasm had no documented infarcts [30].

## 4. Evaluation of Vasospasm in Traumatic Subarachnoid Hemorrhage

Similar to aSAH, vasospasm in tSAH requires a distinction to be made between clinical vasospasm and radiographic or TCD-diagnosed vasospasm, particularly given the lower incidence of clinical vasospasm [8, 11, 30, 39]. Multiple imaging modalities have been utilized to assess cerebral vasospasm. Transcranial Doppler ultrasonography and digital subtraction angiography are the most studied. Following TBI, the incidence of radiographic vasospasm as measured by TCD and catheter angiography is reported to range between 19%–68% [8, 27, 28, 30, 32, 33] and 18.6%–41% [37, 38], respectively. In comparison, the incidence of aSAH-induced vasospasm was noted by TCD to be approximately 38%–45% and by angiography to be 43.2%, on average [34–36] (see Table 1). Noted risk factors include younger age, lower admission GCS, and quantity of cisternal or intracerebral hemorrhage [11, 27, 28]. In a prospective TCD study, Aminmansour et al. demonstrated mild-to-moderate vasospasm in 42.1% of tSAH patients [43]. The authors noted that radiographic vasospasm incidence correlated with extent of injury. In a study of combat victims with TBI, TCD signs of mild, moderate, and severe vasospasms were observed in 37%, 22%, and 12% of patients [14]. Zubkov et al. prospectively assessed 90 head injury patients. TCD ultrasonography demonstrated vasospasm in 35.6% of the study cohort. In accordance with the prior study, these investigators found a significant association between vasospasm and injury severity [8]. A potential correlation between thicker subarachnoid blood on CT scan and degree of vasospasm was also suggested (44.4% versus 31.3%), but it failed to reach statistical significance. Six percent of patients had severe vasospasm, yet only two patients exhibited clinical deterioration, which continues to emphasize the low incidence of clinical vasospasm in tSAH patients. Despite a high prevalence of radiographic vasospasm following tSAH, reliable clinical correlation remains elusive.

Serial TCD ultrasonography protocols have been employed for vasospasm surveillance in the setting of head trauma [30, 31, 44–46]. Noninvasive, relatively inexpensive, and well-validated TCD offers an easy method for serial bedside monitoring [47]. A potential argument against regular surveillance of TBI/tSAH patients derives from the lower incidence of clinically significant vasospasm (compared with aSAH) [8, 11, 30, 39]. However, extrapolation of current data suggests that as many as 279,000 to 1.2 million patients a year may suffer from post-TBI vasospasm in the USA and 10,800 to 40,000 might experience a resultant clinical decline [1, 8, 11, 39]. To this end, advanced physiologic/metabolic imaging techniques such as SPECT have been proposed to assess for areas of decreased perfusion [48]. However, modalities that incorporate radiotracers are expensive and time consuming, and they involve patient exposure to radiation. Although CTA and magnetic resonance angiography are both excellent modalities for detecting vasospasm, their use in surveillance is limited for the same reasons as those of aSAH, namely, expense, difficulty, and, in the case of CT, radiation exposure. If clinical vasospasm is detected, any of these modalities would be appropriate to suggest radiographic vasospasm and prompt treatment. By the same token, DSA would be reserved for detection and treatment of suspected vasospasm, due to either clinical suspicion or diagnosis by TCD. Due to a paucity of data, it is difficult to draw evidence-based conclusions regarding the utility of vasospasm screening in the setting of tSAH.

## 5. Pathophysiology

On cursory evaluation, the pathophysiologies of traumatic and aneurysmal SAH appear similar. The presence of blood in the subarachnoid space, resulting from either head trauma or aneurysmal rupture, results in the narrowing of intracranial vessels. In an early account of traumatic vasospasm, Wilkins and Odom highlight several features that bear similarity to aSAH. They proposed that decrease in vessel caliber is not related to mechanical compression from increased intracranial pressure, but rather to the inherent properties of the extravascular blood products [49]. In this vein, implicated molecular mechanisms like oxyhemoglobin, arachidonic acid, lipid peroxidation, or cortical spreading depression would be present in both etiologies given the presence of blood in both [50–53]. Additionally, Zubkov et al. examined vessel histology following traumatic vasospasm and noted that the morphology appears to be similar to that of aSAH. Both specimens revealed increased connective tissue in the subendothelial layer and diseased internal elastic lamina [39].

However, the difference in timing and severity of tSAH vasospasm coupled with a milder clinical course, and a potential for vasospasm in the absence of cisternal blood, suggests an alternate pathogenesis [30, 54]. These disparities are further supported by the fact that tSAH harbors a distinctly different pattern of ischemia evident on CT scan, rarely correlating with vascular territories [12]. Much of the ischemia seen in tSAH can be explained by contusions and direct injury [12, 55], indicating that vasospasm does not play a role in clinical deterioration and ischemia following TBI. On the other hand, the pattern of SAH in tSAH differs from that in aSAH, occurring more diffusely and with more tentorial hemorrhage [5]. This possibly indicates that the ischemia is due to the presence of blood in small vessel and distal cortical territories: a small-vessel vasospasm.

Multiple mechanisms have been proposed to account for the pathophysiology of vasospasm secondary to tSAH. Mechanical alteration of the cerebral blood vessels, through either direct impact or stretch, has been examined [56–59]. In animal models, Arutiunov et al. demonstrated early onset vasospasm following mechanical vessel manipulation. Vasospasm was evident immediately, but it lasted for only 20 minutes [57]. Similarly, work on blast injury indicates that stretch injuries may be a cause of vasospasm [13, 15, 16]. The blast pressure may be enough to stretch or mechanically alter a vessel causing a vasospasm response. Additionally, blast-induced neurotrauma is another mechanism by which vasospasm can occur in the absence of SAH [13–16]. Such cases may be associated with pseudoaneurysms or other vascular injuries. Much like blast injury from explosives, trauma will cause some degree of shock throughout the central nervous system, resulting in shear and stretch injury. Again, confounding the pathophysiology is the fact that a severe injury is likely to cause more shock to the brain, as well as to result in more SAH.

Still, the anatomic region of greatest hemorrhage density often correlates with the location of vasospasm following tSAH [11, 31, 56, 60], thus suggesting that blood breakdown products may be responsible for the vessel narrowing. The same mechanisms proposed in aSAH, with blood breakdown products and irritation, would exist in both instances. However, this could represent an epiphenomenon, as the traumatic focus and resultant maximal vessel manipulation would also likely coincide with the region of greatest hemorrhage [11, 31, 56, 60]. 

## 6. Prevention and Treatment

Management of tSAH-related vasospasm presents challenges discrete from those encountered in the management of aSAH. The unique critical care issues of tSAH and severe TBI create a distinct challenge for treating vasospasm, where the goals of each therapy can be quite different. For SAH treatment, calcium-channel blockers reduce morbidity following aneurysmal rupture, and the “Triple-H” therapy (hypervolemia, hypertension, and hemodilution) is often initiated to treat vasospasm [19, 26, 61]. These agents, however, could be detrimental in the setting of TBI, depending on the severity of injury and associated comorbidities. Induced hypertension and hypervolemia from the Triple-H therapy can worsen cerebral edema [62, 63], which is a more significant issue in TBI than in aSAH. Additionally, in aSAH, the source of bleeding can be secured through clip ligation, or embolization, and the Triple-H therapy can be done safely with minimal concern for rebleeding. In tSAH, multiple sources of SAH, as well as other sources of intracranial bleeding, like subdural hemorrhages, epidural hemorrhages, and intraparenchymal hemorrhages, make the securing of the sources of bleeding difficult, even with operative intervention. By using the Triple-H therapy, the risk of worsening the hemorrhage is high. In the setting of poly-trauma, the risk of hemorrhage elsewhere in the body is also a considerable concern. Normovolemia has gained popularity in aSAH patients, focusing on hypertension as the most important of the “H's,” moving away from hypovolemia and hemodilution; however, hypertension can still be a serious danger in tSAH patients [64]. Again, rebleeding, in the brain, or elsewhere, is of great concern and would likely offset any gains in vasospasm damage reduction. Calcium-channel blockers can also affect intracranial and cerebral perfusion pressures. Undesirable effects of hypotension are particularly worrisome in TBI patients as hypoperfusion is a noted cause of secondary brain injury [65].

One randomized, controlled, multicenter trial examined the effects of nimodipine treatment (versus placebo) on the incidence of vasospasm (radiographic and clinical) and six-month Glasgow outcome scores in tSAH patients. The authors reported a decrease in cerebral infarction on CT, radiographic vasospasm, and unfavorable clinical outcomes (death, vegetative survival, and severe disability) at six months in the nimodipine group [66]. Another double-blind placebo-controlled trial showed improvement in Doppler flow, but no change in clinical outcome following nicardipine treatment [67]. Other randomized controlled trials have yielded mixed results, although general trends towards clinical improvement are noted [65, 67–70]. However, a Cochrane Review meta-analysis suggested lack of efficacy for calcium-channel blockers in the setting of TBI as a whole. When a subgroup analysis was performed, a small benefit in the patients with TBI and tSAH was demonstrated [71]. A followup pooled analysis of 4 studies conducted exclusively with tSAH patients showed no difference in poor outcome (OR 0.88, 95% CI 0.51–1.54) or mortality (OR 0.95, 95% CI 0.71–1.26) when comparing calcium-channel blocker therapy to placebo treatment [72]. 

Other aSAH therapies that have been translated to treat vasospasm associated with tSAH include intra-arterial papaverine [73], intra-arterial calcium-channel blockers [74, 75], and balloon angioplasty [13, 75, 76]. Reports demonstrate reasonable success, with almost universal resolution of radiographic vasospasm, and often improvements in clinical outcomes and TCD velocities [13, 15]. Similar to aSAH, patients with symptomatic vasospasm following tSAH may benefit from acute intervention for resolution of symptoms, but the use of these interventions in radiographic vasospasm may be less obvious, particularly since the course of vasospasm in tSAH is less severe. The reported data is limited and hinders the ability to draw definitive conclusions.

## 7. Conclusion

Vasospasm following TBI is characterized by a different time course, duration, and associated profile of risk factors than those of aSAH. These discrepancies suggest a potential alternate pathogenesis. The incidence of clinical vasospasm is lower than those of aSAH. However, diagnosis may be limited by decreased mental status and associated injuries/comorbidities. Although surveillance can be performed with relative ease, no treatments have been shown to conclusively affect outcomes. Traumatic subarachnoid patients with severe head injury or large amounts of SAH may benefit from serial noninvasive monitoring and institution of therapy in the setting of radiographic or clinical deterioration. However, further evidence-based evaluation of diagnostic modalities and treatment regimens is warranted.

## Figures and Tables

**Table 1:** Comparison of the epidemiology of traumatic subarachnoid hemorrhage (tSAH) to that of aneurysmal subarachnoid hemorrhage (aSAH). The shorter duration and lower incidence of clinical vasospasm highlight the less severe course of vasospasm following tSAH in comparison to aSAH.

	tSAH	aSAH
Earliest onset (PBD*)	2 [9, 10, 27–30]	3 [8, 26]
Timing of peak vessel-velocity (PBD*)	5–7 [19, 27]	5–14 [19]
Incidence of TCD^†^-diagnosed vasospasm	19%–68% [8, 27, 28, 30, 32, 33]	38%–45% [34–36]
Incidence of angiography-diagnosed vasospasm	18.6%–41% [37, 38]	43.2% [34]
Evidence of clinical vasospasm among those with radiographic vasospasm	3.9%–16.6% [8, 11, 30, 39]	17%–40% [26]

*PBD: postbleed day. ^†^TCD: transcranial Doppler.
